# Automated CT quantification of interstitial lung abnormality in patients with resectable stage I non‐small cell lung cancer: Prognostic significance

**DOI:** 10.1111/1759-7714.15306

**Published:** 2024-04-29

**Authors:** Jae Ho Chung, Jong Myung Park, Do Hyung Kim

**Affiliations:** ^1^ Department of Internal Medicine, International St. Mary's Hospital Catholic Kwandong University College of Medicine Incheon Republic of Korea; ^2^ Department of Thoracic and Cardiovascular Surgery Pusan National University School of Medicine Busan South Korea; ^3^ Department of Thoracic and Cardiovascular Surgery Pusan National University Yangsan Hospital Busan South Korea; ^4^ Transplantation Research Center, Research Institute for Convergence of Biomedical Science and Technology Pusan National University Yangsan Hospital Yangsan South Korea

**Keywords:** computed tomography, deep learning; interstitial lung abnormality, lung cancer

## Abstract

**Background:**

In patients with non‐small cell lung cancer (NSCLC), interstitial lung abnormalities (ILA) have been linked to mortality and can be identified on computed tomography (CT) scans. In the present study we aimed to evaluate the predictive value of automatically quantified ILA based on the Fleischner Society definition in patients with stage I NSCLC.

**Methods:**

We retrospectively reviewed 948 patients with pathological stage I NSCLC who underwent pulmonary resection between April 2009 and October 2022. A commercially available deep learning‐based automated quantification program for ILA was used to evaluate the preoperative CT data. The Fleischner Society definition, quantitative results, and interdisciplinary discussion led to the division of patients into normal and ILA groups. The sum of the fibrotic and nonfibrotic ILA components constituted the total ILA component and more than 5%.

**Results:**

Of the 948 patients with stage I NSCLC, 99 (10.4%) patients had ILA. Shorter overall survival and recurrence‐free survival was associated with the presence of ILA. After controlling for confounding variables, the presence of ILA remained significant for increased risk of death (hazard ratio [HR] = 3.09; 95% confidence interval [CI]: 1.91–5.00; *p* < 0.001) and the presence of ILA remained significant for increased recurrence (HR = 1.96; 95% CI: 1.16–3.30; *p* = 0.012).

**Conclusions:**

The automated CT quantification of ILA, based on the Fleischner Society definition, was significantly linked to poorer survival and recurrence in patients with stage I NSCLC.

## Graphical abstract

Overall survival of patients with and without interstitial lung abnormalities (ILA). The graph shows overall survival (OS) of patients without ILA using Kaplan–Meier estimate. Patients with ILA had significantly shorter OS than those without ILA (*p* < 0.0001).

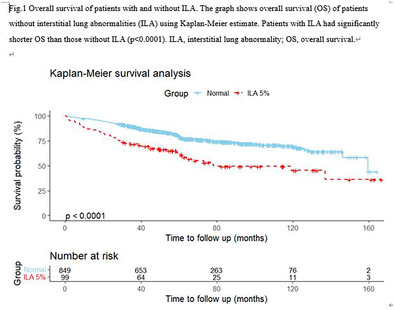

## INTRODUCTION

The clinical significance of incidentally detected interstitial lung abnormalities (ILAs)—defined as incidental computed tomography (CT) findings of nondependent abnormalities affecting >5% of any lung zone (i.e., upper, middle, and lower lung zones demarcated by the levels of the inferior aortic arch and right inferior pulmonary vein) on complete or partial chest CT, where interstitial disease was not previously suspected^1^—is becoming more widely recognized. The prognostic significance of ILAs has recently been emphasized.^2^ Lung cancer screening cohort studies^2^, ^3^ have found ILA in 8%–10% of lung cancer screening participants. ILAs have been associated with all‐cause mortality,^3^ elevated risk of lung cancer diagnosis, and lung cancer mortality.^4^ In addition, the significance of ILAs has been investigated in patients with treatable lung cancer. According to Im et al.,^5^ ILA has a negative effect on lung cancer prognosis, with a 5‐year mortality rate that is 2.6 times higher than that of non‐ILA. According to Iwasawa et al.,^6^ disease‐free survival is linked to the automated assessment of the number of ILA in preoperative CT scans for lung cancer.

ILA interpretation by radiologists is subjective; therefore, when assessing ILA, reproducible interpretation using objective methods is necessary. The clinical importance of ILA detection has been increasingly emphasized. However, proper and reproducible ILA identification can be challenging, considering that ILAs are defined as nondependent abnormalities affecting >5% of any lung zone (i.e., upper, middle, and lower lung zones demarcated by the levels of the inferior aortic arch and right inferior pulmonary vein), with the 5% threshold subject to high variability among different readers.^7^ The adoption of a quantification system is one method to solve these problems.^7^ Deep learning‐based automated quantification systems can be used to identify ILA, based on the Fleischner Society criteria, while simultaneously identifying the subcategories in each lung zone.

We hypothesized that a correlation would exist between ILA and death in patients with stage I non‐small cell lung cancer (NSCLC). Thus, our goal was to evaluate the prognostic value of automatically quantified ILA diagnoses, based on the Fleischner Society definition, in patients with stage I NSCLC.

## METHODS

### Study participants

This study included 948 patients who underwent lobectomy with curative intent for stage 1 NSCLC between April 2009 and October 2022 (American Joint Committee on Cancer [AJCC] 8th edition) diagnosed between 2009 and 2022 at Pusan Yangsan Hospital were obtained from the institute's lung cancer case file. These files contained pre‐ and postoperative clinical data and analyzable preoperative clinical records of demographics such as age, sex, smoking status, and chest CT within 90 days after surgery.

The Institutional Review Board approved this retrospective investigation and waived the requirement for signed informed consent (IRB no. 55‐2023‐069). Potential prognostic factors were extracted from comorbidities (e.g., diabetes mellitus, hypertension, respiratory diseases, cardiovascular diseases, cerebrovascular disease, liver dysfunction, and renal dysfunction), previous cancer history, spirometry tests, body mass index, and surgical pathology reports. Demographics (e.g., age and sex), smoking status (nonsmoker vs. ex‐smoker or current smoker), and other information were obtained from electronic medical records.

### Chest CT acquisition

Chest CT was performed by using multidetector row CT scanners from two manufacturers (Siemens Healthineers and GE Healthcare). The acquisition parameters were 120 kVp, 30–200 mAs, a pitch of 0.875–1, and a collimation of 1–1.25 mm. Intravenous contrast medium (90–120 mL) was injected at a rate of 3 mL/s and the scan delay was 50 s. Images were reconstructed using a sharp kernel with a slice thickness/interval of 1/1 mm or 1.25/1.25 mm.

### Automatic quantification of ILA

Commercially available deep learning‐based automated quantification software for ILA (Aview, version 1.1.38.6; Coreline Soft) was used to evaluate the whole‐lung images of all chest CT scans. This tool performs fully automatic segmentation of the total lung parenchyma and each finding (i.e., emphysema, ground‐glass opacity [GGO], consolidation, reticular opacity, and honeycombing cysts) on the input CT images, and provides quantification results as percentages (%) of the total lung volume. We extracted reticular opacity, GGO, and honeycombing cysts as interstitial lung disease (ILD) CT findings. The lung parenchyma was segmented into each lung zone, based on anatomical landmarks (i.e., the levels of the inferior aortic arch and the right inferior pulmonary vein), as indicated by the definition of ILA, and were then divided into three categories: (1) normal parenchyma; (2) fibrotic ILA component equal to the quantified extent of honeycombing (including architectural distortion and traction bronchiectasis); and (3) nonfibrotic ILA component (i.e., the sum of the quantified extent of GGO and reticulation).^7^ The total ILA component is the sum of the fibrotic and nonfibrotic ILA components. The fractional results of each category, total ILA components, and number of affected lung zones were determined. No additional manual modifications were made to segmentation results. Changes in each ILD finding, fibrosis extent, and total ILD extent between the initial and follow‐up CT images were calculated. The segmentation performance of the software was substantially consistent with that of thoracic radiologists.^8^

### Classification of ILA with definition by the Fleischner Society

Based on the Fleischner Society criteria,^7^ total ILA is the sum of the nonfibrotic and fibrotic ILA values. The system finally identified ILA on chest CT when the maximum extent of the total ILA was at least 5% in any of the six zones and highlighted positive results on the user interface. The sum of the fibrotic and nonfibrotic ILA components was considered the total ILA component, and >5% was defined as the ILA group.

### Follow‐up for survival analysis

Institutional electronic medical records were used to gather the patients' data. Survival analysis included two endpoints: recurrence‐free survival (RFS) and overall survival (OS). The time from surgery to recurrence or death from any cause (i.e., event) or until the patients were free of NSCLC recurrence was defined as RFS. The time from surgery to death from any cause or until the patient was last known to be alive was defined as the OS. The data were archived until March 2023. Data were censored when the information about survival time was incomplete and most right‐censoring data were lost to follow‐up.

### Statistical analysis

The *t*‐test was used to compare continuous variables, whereas Pearson's chi‐square or Fisher's exact tests were used to compare categorical data between the two groups (i.e., normal and ILA). The Kaplan–Meier method was used to generate survival curves, stratified by ILA based on the Fleischner Society definition, and were then compared using the log‐rank test. Univariate and multivariate Cox proportional hazards regression analyses were used to determine the prognostic markers for RFS and OS in the complete population of patients with stage I NSCLC. All statistical analyses and graphics were created using R programming language and a variety of tools. Statistical significance was set at *p* < 0.05.

## RESULTS

Of the 948 patients included in the study (mean age, 66.3 ± 8.8 years; age range, 15–86 years; 561 men), 849 (89.6%) patients were categorized as normal, based on the Fleischner Society definition and 99 (10.4%) patients had ILAs. Table 1 summarizes the demographics of the patients, illness features of the study group, and baseline ILA status on chest CT. The median follow‐up time was 32.2 months (range, 0.4–169.0 months). Patients with ILAs were significantly older, had more liver impairment, and had lower diffusing capacity of the lungs for carbon monoxide (DL_CO_). The median [range] follow‐up duration for RFS was 59.5 [0.4–169.0] months and for OS was 60.3 [0.4–166.6] months. A total of 295 overall recurrences occurred, as well as 123 events and 24 events in the normal and ILA groups, respectively. Of the 243 deaths, 198 and 45 occurred in the control and ILA groups, respectively.

**TABLE 1:** Clinical characteristics of study populations.

Patient characteristics	Total (*N* = 948)	Normal (*N* = 849)	ILA (*N* = 99)	*p*‐value
Age (years)	66.3 ± 8.8	65.8 ± 8.9	70.4 ± 6.6	0.001
Sex				0.332
Female	387 (40.8%)	342 (40.3%)	45 (45.5%)	
Male	561 (59.2%)	507 (59.7%)	54 (54.5%)	
BMI, kg/m^2^	23.9 ± 3.0	23.8 ± 3.0	24.8 ± 3.3	0.210
Smoking				0.111
Non‐smoker	471 (49.7%)	414 (48.8%)	57 (57.6%)	
Ex/current smoker	477 (50.2%)	435 (51.2%)	42 (42.4%)	
Respiratory disease	95 (10.0%)	82 (9.7%)	13 (13.1%)	0.288
Cerebrovascular disease	60 (6.3%)	53 (6.2%)	7 (7.1%)	0.666
Cardiovascular disease	109 (11.5%)	95 (11.2%)	14 (14.1%)	0.404
Liver dysfunction	31 (3.3%)	23 (2.7%)	8 (8.1%)	0.011
Renal dysfunction	21 (2.2%)	19 (2.2%)	2 (2.0%)	1.000
Hypertension	377 (39.8%)	333 (39.2%)	44 (44.4%)	0.330
DM	208 (21.9%)	178 (21.0%)	30 (30.3%)	0.040
Previous cancer history	175 (18.5%)	156 (18.4%)	19 (19.2%)	0.891
FEV_1_ (% pred)	87.8 ± 16.9	87.6 ± 16.9	89.4 ± 17.3	0.313
FVC (% pred)	89.4 ± 15.6	87.6 ± 16.9	89.4 ± 17.3	0.016
FEV_1_/FVC (% pred)	71.1 ± 10.7	70.8 ± 10.7	72.7 ± 10.3	0.294
DL_CO_ (% pred)	87.5 ± 19.3	88.1 ± 19.2	83.2 ± 19.7	0.027
Pathological type				0.374
ADC	716 (75.5%)	646 (76.1%)	70 (70.7%)	
SCC	189 (19.9%)	164 (19.3%)	25 (25.3%)	
Others	43 (4.5%)	39 (4.6%)	4 (4.0%)	

Abbreviations: ADC, adenocarcinoma; BMI, body mass index; DL_CO_, diffusing capacity of the lung for carbon monoxide; DM, diabetes mellitus; FEV_1_, forced expiratory volume in 1 second; FVC, forced vital capacity; ILA, interstitial lung abnormality; SCC, squamous cell cancer.

**FIGURE 1:**
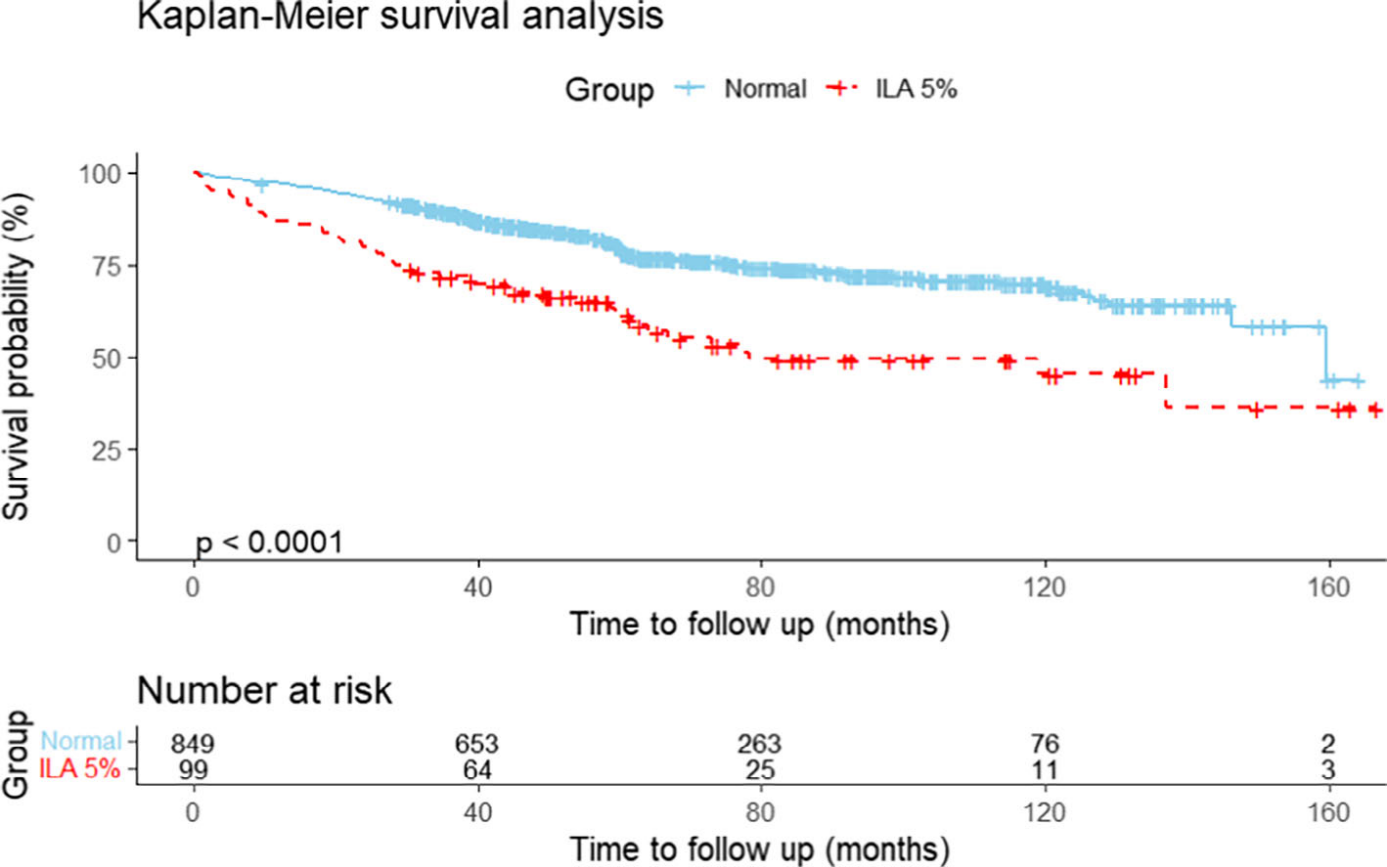
Overall survival (OS) of patients with and without interstitial lung abnormalities (ILA). The graph shows OS of patients without ILA using Kaplan–Meier estimate. Patients with ILA had significantly shorter OS than those without ILA (*p* < 0.0001).

**FIGURE 2:**
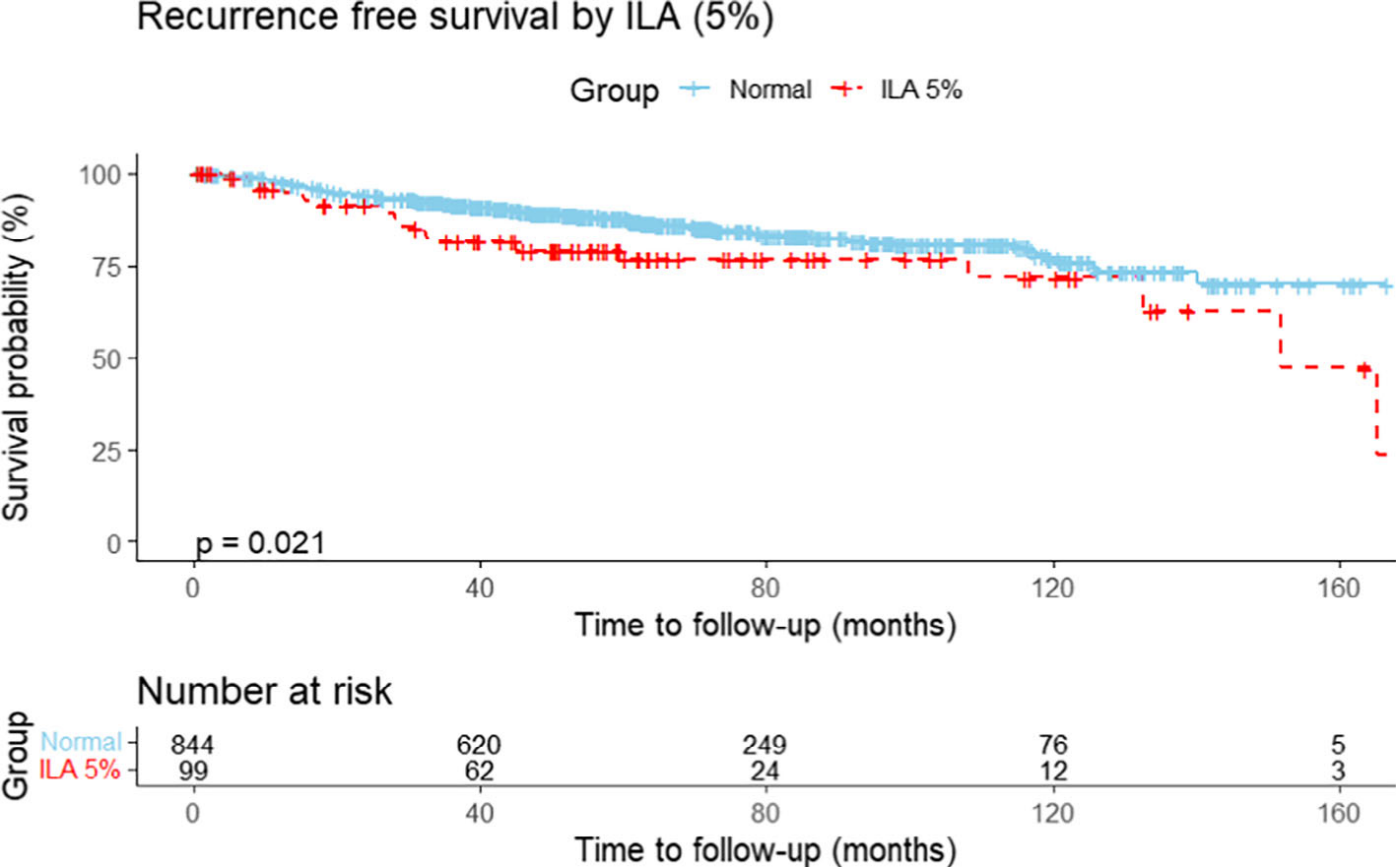
Recurrence‐free survival (RFS) of patients with and without interstitial lung abnormalities (ILA). The graph shows overall disease‐free survival (DFS) of patients without ILA using Kaplan–Meier estimate. RFS was significantly shorter in patients with ILA than non‐ILA group (*p* = 0.021).

In the Cox proportional hazards model, the univariate and multivariate hazard ratios (HRs) of the risk factors associated with OS are shown in Table 2, and RFS is shown in Table 3. After controlling for confounding variables, the presence of ILA remained significant for increased risk of death (HR = 3.09; 95% confidence interval [CI], 1.91–5.00; *p* < 0.001) and the presence of ILA remained significant for increased recurrence (HR = 1.96; 95% CI, 1.16–3.30; *p* = 0.012). The presence of ILA was associated with shorter OS and the median OS of the 99 patients with ILA was mean 95.5 months [95% CI: 80.8–110.3] compared to mean 125.3 months [95% CI: 120.3–130.3] in patients with normal group (*p* < 0.0001, Figure 1). RFS was substantially shorter in the ILA group, with a mean of 126.1 months (95% CI, 111.3–140.9), than in the normal group, with a mean of 138.9 (95% CI: 134.2–143.7) months (*p* = 0.021, Figure 2).

**TABLE 2:** Univariable and multivariable hazard ratio of risk factors associated with overall survival in the Cox proportional hazard model.

Risk factors	Univariable HR (95% CI)	*p*‐value	Multivariable HR (95% CI)	*p*‐value
Sex				
Female	Reference		‐	‐
Male			2.16 (1.35–3.45)	0.001
Age, (years)	1.06 (1.04–1.08)	<0.001	1.04 (1.02–1.06)	<0.001
BMI, kg/m^2^	0.88 (0.83–0.92)	<0.001	0.87 (0.82–0.92)	<0.001
Ex/current smoker	2.30 (1.70–3.12)	<0.001	1.22 (0.80–1.86)	0.352
Respiratory disease	2.00 (1.29–3.12)	0.002	1.50 (0.92–2.45)	0.103
Cerebrovascular disease	1.26 (0.71–2.24)	0.424	0.94 (0.50–1.75)	0.936
Cardiovascular disease	1.51 (0.99–2.31)	0.061	1.17 (0.73–1.88)	0.502
Liver dysfunction	1.62 (0.77–3.44)	0.206	1.25 (0.55–2.85)	0.600
Renal dysfunction	1.81 (0.74–0.4.43)	0.192	1.31 (0.50–3.39)	0.585
Hypertension	1.30 (0.96–1.74)	0.085	1.21 (0.86–1.71)	0.277
DM	1.23 (0.88–1.74)	0.230	1.00 (0.68–1.48)	0.986
Previous cancer history	1,43 (1.00–2.04)	0.049	1.36 (0.90–2.00)	0.143
Pathological type				
ADC	Reference			
SCC	2.87 (2.04–4.03)	<0.001	1.57 (1.06–2.32)	0.025
Others	2.55 (1.35–4.83)	0.004	2.12 (1.03–4.32)	0.040
ILA (5%)	2.74 (1.79–4.20)	<0.001	3.09 (1.91–5.00)	<0.001

Abbreviations: ADC, adenocarcinoma; BMI, body mass index; CI, confidence interval; DM, diabetes mellitus; HR, hazard ratio; ILA, interstitial lung abnormality; OS, overall survival; SCC, squamous cell cancer.

**TABLE 3:** Univariable and multivariable hazard ratio of risk factors associated with recurrence‐free survival in the Cox proportional hazards model.

Risk factors	Univariable HR (95% CI)	*p*‐value	Multivariable HR (95% CI)	*p*‐value
Sex				
Female	Reference		‐	‐
Male			1.32 (0.78–2.24)	0.302
Age, (years)	1.02 (1/00–1/04)	0.042	1.00 (0.9–1.03)	0.557
BMI, kg/m^2^	0.97 (0.92–1.03)	0.395	0.96 (0.90–1.02)	0.205
Ex/current smoker	1.54 (1.08–2.20)	0.018	1.25 (0.76–2.04)	0.379
Respiratory disease	1.54 (0.91–2.61)	0.111	1.36 (0.79–2.04)	0.271
Cerebrovascular disease	1.43 (0.64–3.20)	0.399	1.73 (0.75–3.96)	0.788
Cardiovascular disease	1.09 (0.64–1.89)	0.743	1.08 (0.62–1.88)	0.738
Liver dysfunction	1.31 (0.53–3.26)	0.558	1.14 (0.45–2.87)	0.789
Renal dysfunction	2.38 (0.90–6.29)	0.081	2.20 (0.82–5.95)	0.107
Hypertension	1.56 (1.11–2.25)	0.011	1.62 (0.53–1.31)	0.015
DM	1.08 (0.71–1.64)	0.733	1.21 (0.77–1.90)	0.404
Previous cancer history	1.19 (0.74–1.91)	0.470	1.27 (0.78–2.07)	0.330
Pathological type				
ADC	Reference			
SCC	1.43 (0.95–2.17)	0.090	1.18 (0.47–2.95)	0.726
Others	0.95 (0.39–2.30)	0.906	1.23 (0.47–3.23)	0.675
ILA (5%)	1.88 (1.4–3.09)	0.013	1.96 (1.16–3.30)	0.012

Abbreviations: ADC, adenocarcinoma; BMI, body mass index; CI, confidence interval; DM, diabetes mellitus; HR, hazard ratio; ILA, interstitial lung abnormality; RFS, recurrence‐free survival; SCC, squamous cell cancer.

## DISCUSSION

In our study, both disease categories, based on the recent definition and automated quantification of ILA components in stage I NSCLC, were identified as independent risk factors for poor RFS and OS. Given the necessity of evaluating each zone for ILA, visually assessing the proportion of the ILA in each zone on two‐dimensional CT scans may be more difficult. Given the different prognoses, based on the disease category in patients with resectable stage I NSCLC, reliable and reproducible identification of ILA with discrimination is important. One possible solution is the implementation of an automated ILA quantification system. The Fleischner Society recently proposed a revised definition of ILA and related terminology for standardization.^7^ ILA has been linked to the increased incidence and mortality of lung cancer.^4^ Furthermore, ILA is linked to a higher likelihood of postoperative problems after lung cancer surgery, radiation, and immunotherapy.^9^, ^10^ In our study, the automated quantification system clarified the extent of ILA components in each zone with the total number of zones with ILA involvement of ≥5%.

Although a few previous studies have demonstrated that ILAs are associated with NSCLC prognosis, our analysis revealed that ILAs were a significant risk factor for poor prognosis in patients with stage I NSCLC, which is consistent with the findings of previous research.^6^, ^11^, ^12^ Tomoyuki et al.^11^ demonstrated that the presence of ILA remained significantly associated with an increased risk of death (HR = 2.88, *p* = 0.005) in 231 patients with stage I NSCLC. Our study showed that, compared with the normal group, patients with ILA had a higher HR (3.09) OS. Iwasawa et al.^6^ studied 217 patients with stage I and II NSCLC and discovered that the presence of ILA was associated with shorter RFS (HR, 3.3; *p* < 0.001). Im et al.^12^ studied 488 patients who underwent curative resections for stage I and II NSCLC and discovered that ILAs were independently associated with postoperative pulmonary complications; four patients died 180 days after surgery because of respiratory failure, aspiration pneumonia, and empyema. Putman et al.^7^ found that participants with ILA were more likely to die of respiratory causes than were patients without ILA (HR, 2.4; *p* < 0.001). A Chinese study^13^ also demonstrated that the presence of ILA in patients with NSCLC was significantly associated with a shorter OS than that of patients without ILA (751 days vs. 445 days, HR 0.6, *p* = 0.001). In patients with treatment‐naïve advanced NSCLC with ILA, the median OS was significantly shorter than that of patients without ILA (median OS: 7.2 months vs. 14.8 months; *p* = 0.002).^14^ Two other recent studies also support that ILA is associated with shorter overall survival in patients with stage I NSCLC (median OS: 46.2 months vs. 121.92 months; *p* < 0.0001) and stage IV NSCLC (median OS: 9.95 months vs. 16.95 months; *p* = 0.0002).^11^, ^15^ Moreover, the presence of ILA alone was a prognostic factor for postoperative pulmonary complications in lung cancer (adjusted HR, 1.91; *p* = 0.004), which is the major cause of perioperative morbidity and mortality after lung resection.^12^

Our study revealed that patients with stage I NSCLC with ILA had a shorter RFS than did patients with stage I NSCLC without ILA. This finding is consistent with the results of previous studies.^6^, ^16^ The presence of ILA was a predictor of poorer disease‐free survival in patients with lung cancer in studies by Iwasawa et al.^6^ (HR, 3.3; 95% CI: 1.8–6.2; *p* = 0.001) and Ahn et al.^16^ (HR, 1.81; 95% CI: 1.25–2.61; *p* = 002), which suggest that patients who have lung cancer with ILA have an increased risk of early death owing to lung cancer. Although our study demonstrated a prognostic significance with regard to RFS and OS, a large, well‐designed prospective study will elucidate this issue to understand why individuals with ILA have a lower survival rate and poorer RFS among those with NSCLC.

Our study had several limitations. First, the participants in this study were treated at a single site, the approach was retrospective, and missing data introduced a natural selection bias. A larger cohort is required to validate the findings of this study. Second, the utilization of multiple vendors, nonenhanced high‐resolution CT, and the inability to perform scans in the prone position meant that the CT scan was not specifically intended for automated measurement of ILA.

Automated CT quantification scans may be useful because ILAs are inadvertent anomalies found on CT scans when using diverse methods in real‐world practice. Iwasawa et al.^6^ also employed chest CT with a soft tissue kernel to quantify the ILA. Third, the currently available software does not distinguish nonemphysematous cysts from emphysema, which by definition includes fibrotic ILA. Finally, the results of this single‐center study inevitably have limited generalizability; therefore, additional external validation would be beneficial.

In conclusion, automated CT quantification of ILAs, based on the Fleischner Society criteria, predicts the outcomes of patients with stage I NSCLC. This study adds to the data supporting the idea that ILAs are associated with important clinical outcomes in stage I NSCLC and suggests a path toward refining ILA by stratifying patients at the greatest risk of experiencing adverse clinical outcomes.

## AUTHOR CONTRIBUTIONS

All authors contributed to the conception, analysis, interpretation, revision, and final preparation of the manuscript. Do Hyung Kim served as the principal investigator and had full access to all study data. Jong Myung Park and Jae Ho Chung take responsibility for the integrity of the data and accuracy of the data analysis.

## FUNDING INFORMATION

This work was supported by a 2‐year research grant from Pusan National University.

## CONFLICT OF INTEREST STATEMENT

None of the authors has any conflicts of interest.
